# The Threatened Species Imperative: Conservation assessments would benefit from population genomic insights

**DOI:** 10.1073/pnas.2210685119

**Published:** 2022-08-15

**Authors:** J. Andrew DeWoody, Jong Yoon Jeon, John W. Bickham, Erangi J. Heenkenda, Safia Janjua, Gina F. Lamka, Andrew J. Mularo, Andrew Black, Anna Brüniche-Olsen, Janna R. Willoughby

**Affiliations:** ^a^Department of Forestry and Natural Resources, Purdue University, West Lafayette, IN 47907-1159;; ^b^Department of Biological Sciences, Purdue University, West Lafayette, IN 47907;; ^c^Department of Ecology and Conservation Biology, Texas A&M University, College Station, TX 77843;; ^d^College of Forestry, Wildlife, and Environment, Auburn University, Auburn, AL 36849;; ^e^Department of Biology, Section for Computational and RNA Biology, University of Copenhagen, 2200 Copenhagen, Denmark

**Fig. 1.**
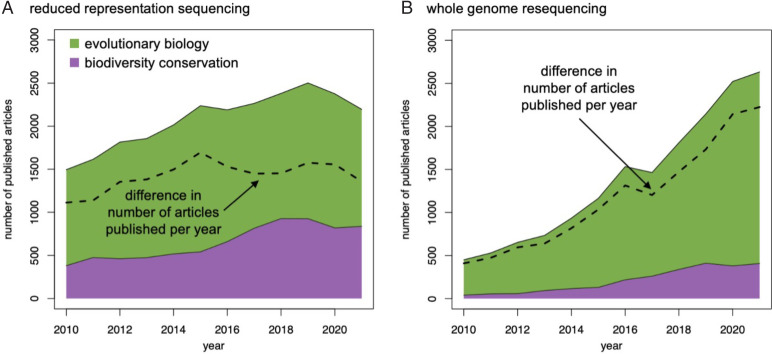
Publishing trends indicate that papers in evolutionary biology (green) are transitioning from reduced representation to whole-genome resequencing; those in conservation biology (purple) lag behind in this technological transition. Data are from the Institute for Scientific Information's Web of Science.

Hogg et al. (1) recently described the Threatened Species Initiative they designed to help apply genomic data to conservation decisions. We applaud and support such efforts, as genetic/genomic diversity (GD) is a key aspect of biodiversity that should be explicitly incorporated into conservation ranking priorities (2, 3). Hogg et al. (1) argue that assessments of GD based on reduced representations of the genome are desirable because they 1) are cost-effective, 2) can be used with suboptimal sources of DNA, and 3) can provide fundamental biological insights (e.g., population subdivision) that can empower nongeneticists who are integral to conservation efforts. We generally agree. However, we note that the economic advantages of reduced representation are rapidly diminishing or even nonexistent (4), and its application is already decreasing in fields like evolutionary biology, where whole-genome resequencing is supplanting reduced representation (Fig. 1).

What is the growing appeal of whole-genome resequencing datasets? Whole-genome resequencing allows practitioners to efficiently parse GD into neutral and functional components (e.g., in protein-coding genes of known or suspected function), whereas most reduced representation sequencing occurs in putatively neutral regions. GD at neutral sites is useful for estimating important parameters, such as effective population size or migration rates (5), but these values alone cannot fully describe population genomic attributes. The variation found in protein-coding genes can identify signatures of natural selection (e.g., the evolutionary underpinnings of local adaptation) that have important management implications. For example, sage-grouse show patterns of local adaptation related to their ability to remove toxins from sagebrush, a major food source that does not occur uniformly across the species’ range. Effective management of this species thus requires consideration of these environmental and genomic variants to prevent fitness reductions detrimental to conservation efforts (6).

Whole-genome resequencing also allows assessments of parameters that may influence population growth rates. Inbreeding can be directly quantified from whole-genome resequencing (e.g., runs of homozygosity) and used to discern how biological and environmental factors shape GD over time (7). Parameters such as genetic load can also have large effects on population growth rates by altering the number of young that are produced, survive, and ultimately, reproduce (8, 9). Furthermore, whole-genome resequencing data can provide key insights into hybridization and associated tracts of introgression (10).

We commend Hogg et al. (1) for their Threatened Species Initiative and hope it flourishes for the benefit of conservation worldwide. Our field is expanding far beyond simple empirical descriptions of GD, and given the ongoing biodiversity crisis and with respect to conservation assessments, we think that whole-genome resequencing data will soon become a key pillar of what could be called a “Threatened Species Imperative.”
